# F247. INTERNALIZED STIGMA HAS A STRONGER RELATIONSHIP WITH INTRINSIC MOTIVATION COMPARED TO AMOTIVATION IN EARLY PHASE AND PROLONGED SCHIZOPHRENIA

**DOI:** 10.1093/schbul/sby017.778

**Published:** 2018-04-01

**Authors:** Ruth Firmin, Lauren Luther, Paul Lysaker, Jennifer Vohs

**Affiliations:** 1 UCLA; 2 Indiana University, Purdue University; 3 Roudebush VA Medical Center, Indiana University School of Medicine; 4 Indiana University School of Medicine

## Abstract

**Background:**

Motivation deficits predict decreased functioning in schizophrenia. Recent work suggests deficits reflect challenges in separate domains: intrinsic motivation (one’s internal drive to engage in a behavior out of enjoyment or interest) and amotivation (one’s broader decrease in motivated behavior linked to avolition and anhedonia). Internalized stigma is another determinant of functioning for people with schizophrenia that may impact motivation. However, little is known about these relationships, including which aspects of motivation it may impact nor when these links emerge. Identifying the link between these constructs may help to identify whether internalized stigma may be a novel treatment target to facilitate improvements in motivation.

**Methods:**

Forty adults with early phase schizophrenia and 66 adults with prolonged schizophrenia completed measures of internalized stigma, intrinsic motivation, and amotivation. Pearson’s correlations were examined followed by Fischer’s r-to-z transformations to compare differences in the magnitude of associations between internalized stigma and intrinsic motivation and internalized stigma and amotivation among the first episode and prolonged samples. Next, we conducted stepwise regressions to examine whether internalized stigma was associated with intrinsic motivation above and beyond associations with amotivation in each sample.

**Results:**

In the early phase sample, the association between internalized stigma was greater with intrinsic motivation (r=-0.48, p=.00) compared to amotivation (r=0.27, p=0.10). Associations with internalized stigma in the prolonged sample were also greater with intrinsic motivation (r=-0.30, p=0.02) versus amotivation (r=0.19, p=0.12). The magnitude of the associations between internalized stigma and intrinsic motivation (z=1.03, p=0.15) and between internalized stigma and amotivation (z=0.41, p = 0.34) did not significantly differ when comparing phase of illness. Regression analyses indicated that, controlling for amotivation, internalized stigma predicted intrinsic motivation in both the prolonged sample (R2=0.09, F(1,64) =6.18, p=0.02) and the early phase schizophrenia sample (R2=0.23, F(1,37)=10.98, p=.00).

**Discussion:**

Results suggest internalized stigma has a stronger relationship with intrinsic motivation separate from, and above and beyond, its association with amotivation. Findings support models of intrinsic and amotivation being distinct domains. Links between internalized stigma and motivation appear to emerge and persist from the early stages of schizophrenia, suggesting that targeting stigma in early intervention services may help to improve intrinsic motivation in people with schizophrenia.

